# Meetings of Learned Bodies

**Published:** 1851-04

**Authors:** 


MEETINGS OF LEARNED BODIES.

It has been published incorrectly several times that the American Medical Association meets on the 2d Tuesday in May, instead of the *first* Tuesday. Charleston, the place of meeting, has long enjoyed a high reputation for the elegant hospitality of its inhabitants, and we feel quite sure that they will do their part towards rendering the next meeting one of the most pleasant that this learned body has held. When the American Association for the Advancement of Science met there a year ago, the City Council came forward and defrayed the expense of the meeting, and in addition to this, we believe, the members who traveled by railroads were not charged for their conveyance going or returning.

Cincinnati is now emulating the noble example of Charleston. The next meeting of the American Association for the Advancement of Science takes place, in that city, on Monday, the 5th of May, and from the Circular of the Secretary, which we have just received, we perceive that the local committee of the Association have "provided for the gratuitous entertainment of all members attending."

These facts speak well for the liberality and intelligence of those cities. In honoring science they do honor to themselves. Both meetings in May are sure to be large, for men of cultivation and refinement are naturally drawn towards communities where the objects of their tastes and affections are duly appreciated. We can hardly imagine anything more agreeable than to be present at the deliberations and discussions of either of these bodies, and we would advise all who have it in their power to attend.

